# DNA-Free Recombinant SV40 Capsids Protect Mice from Acute Renal Failure by Inducing Stress Response, Survival Pathway and Apoptotic Arrest

**DOI:** 10.1371/journal.pone.0002998

**Published:** 2008-08-20

**Authors:** Veronika Butin-Israeli, Dotan Uzi, Mahmoud Abd-El-Latif, Galina Pizov, Arieh Eden, Yosef S. Haviv, Ariella Oppenheim

**Affiliations:** 1 Department of Hematology, Hebrew University-Hadassah Medical School, Jerusalem, Israel; 2 Department of Pathology, Hebrew University-Hadassah Medical School, Jerusalem, Israel; 3 Department of Anesthesiology and Critical Care Medicine, Carmel Lady Davis Medical Center, Haifa, Israel; 4 Department of Nephrology, Hebrew University-Hadassah Medical School, Jerusalem, Israel; University of Giessen Lung Center, Germany

## Abstract

Viruses induce signaling and host defense during infection. Employing these natural trigger mechanisms to combat organ or tissue failure is hampered by harmful effects of most viruses. Here we demonstrate that SV40 empty capsids (Virus Like Particles-VLPs), with no DNA, induce host Hsp/c70 and Akt-1 survival pathways, key players in cellular survival mechanisms. We postulated that this signaling might protect against organ damage *in vivo*. Acute kidney injury (AKI) was chosen as target. AKI is critical, prevalent disorder in humans, caused by nephrotoxic agents, sepsis or ischemia, via apoptosis/necrosis of renal tubular cells, with high morbidity and mortality. Systemic administration of VLPs activated Akt-1 and upregulated Hsp/c70 *in vivo*. Experiments in mercury-induced AKI mouse model demonstrated that apoptosis, oxidative stress and toxic renal failure were significantly attenuated by pretreatment with capsids prior to the mercury insult. Survival rate increased from 12% to >60%, with wide dose response. This study demonstrates that SV40 VLPs, devoid of DNA, may potentially be used as prophylactic agent for AKI. We anticipate that these finding may be projected to a wide range of organ failure, using empty capsids of SV40 as well as other viruses.

## Introduction

Simian virus 40 (SV40) is a non-enveloped primate virus, with a small, double-stranded, circular DNA genome of 5.2 kb. SV40 infects dividing and non-dividing cells and does not depend on host cell cycle [1]. This is in contrast to many other viruses that infect only dividing cells and enter the nucleus during mitosis when the nuclear envelope is disassembled. This suggests that SV40 activates signaling pathways that permit nuclear entry of the viral genome. SV40 enters host cells by an atypical, slow endocytic process mediated by caveolae [2], [3] via the endoplasmic reticulum [4]. This is unlike most viruses, that utilize clathrin-coated and non-coated vesicles for endocytosis [5]. In a parallel project we have started a detailed study on cellular signals induced by the infecting SV40. We have found that SV40 activates Akt-1 survival pathway and the Hsp/c70 chaperones via PLC-γ signaling (unpublished results), very early post infection. SV40 also protected CV-1 cells against etoposide-induced apoptosis (our unpublished results). These findings led us to hypothesize that cellular uptake of SV40, or the viral capsid alone, might function in protection against *in vivo* cellular damage and degenerative diseases. Because SV40 has a natural affinity to the kidney, we chose renal failure as a first target for testing the hypothesis.

Acute kidney injury (AKI), previously known as acute tubular necrosis (ATN), is characterized by abrupt and reversible kidney dysfunction, caused by sepsis, ischemia or nephrotoxic agents. The major underlying pathogenic mechanisms are apoptosis and necrosis of kidney tubular cells [6], in particular in the proximal convoluted tubuli. Many recent studies suggest that chaperones, in particular members of the Hsp70 family, may provide protection against AKI [7] (for recent reviews see [8], [9], [10]).

Nephrotoxic agents are commonly used in medicine. These include contrast material that is administered routinely to patients undergoing radiological procedures, such as computerized tomography and cardiac and other angiographies. Contrast media induced nephropathy is the third most common cause of acute renal failure in hospitalized patients, and may lead to the development of end stage acute renal failure in up to 10% of patients with prior renal dysfunction, and also in patients with prior normal renal function [11], [12]. Furthermore, in patients who develop contrast media induced nephropathy following coronary interventions, long term mortality is increased irrespective of the presence of prior renal dysfunction [13]. In addition, various drugs, in particular anticancer, antibiotic and immunosuppressant drugs are also nephrotoxic. Patients who are exposed to nephrotoxic agents may be subjected to additional insults to the kidney, such as sepsis, hypoperfusion, or ischemia and reperfusion. These multiple insults increase the risk for AKI. AKI is associated with high morbidity and mortality and is a major economic burden due to the high cost of supportive medical treatment. The mortality among critically ill patients with AKI remains high despite increasing ability to support vital organs [14]. Thus, any therapeutic modality conferring resistance to AKI is anticipated to have a large impact on improved survival of critically ill patients and on public health.

Recombinant SV40 capsids are readily produced in *Spodoptera frugiperda* (Sf9) cells using recombinant baculovirus expressing VP1 from the polyhedrin promoter [15], [16]. VP1 spontaneously assemble in the nuclei of infected Sf9 cells forming Virus-Like-Particles (VLPs). Purified VLPs appear as isolated nanoparticles of uniform shape and size similar to wild type SV40 virions (**Figure S1**). Gel electrophoresis experiments indicate that they contain >95% VP1.

## Results

### SV40 VLPs upregulate Hsp/c70 and activate Akt-1 in monkey kidney cells

**Figure 1:**
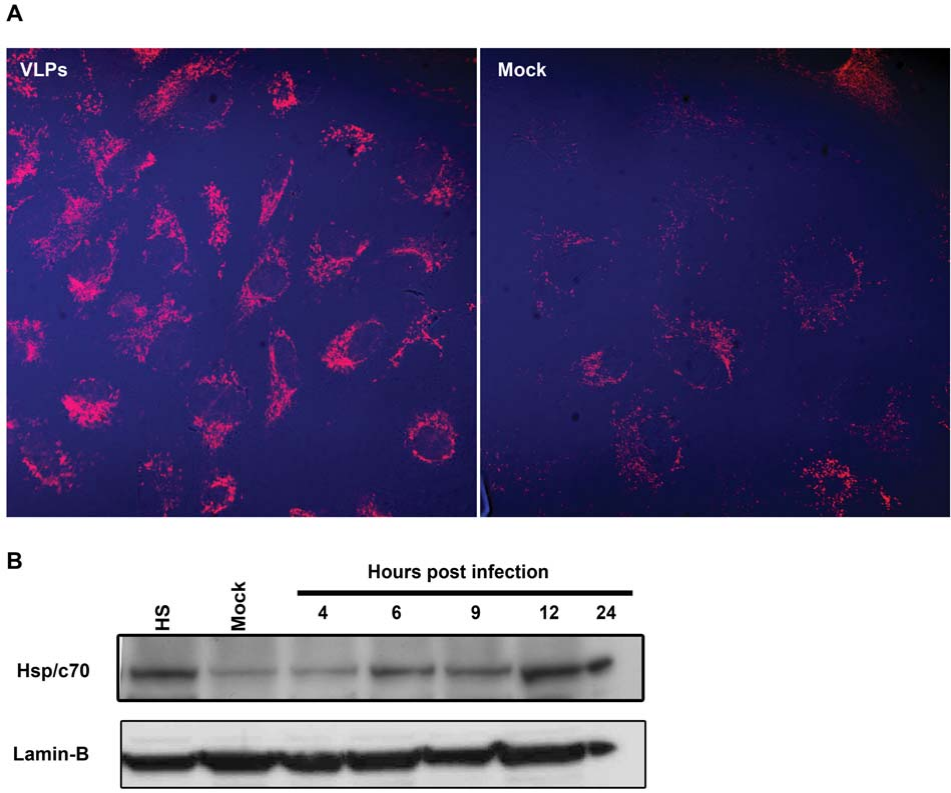
Upregulation of Hsp/c70 in monkey cultured kidney cells. CV-1 cells were treated with 50 ng VLPs per 10^6^ cells, equivalent to moi of 10 of wild type SV40. (A) Hsp/c-70 analyzed by confocal microscopy at 9 hours post treatment with VLPs. Left-VLP-treated cells; Right–Mock treated cells. (B) Hsp/c-70 analyzed by Western blotting with monoclonal antibody against Hsp/c70 at the designated time points. HS–heat-shocked cells serving as a positive control for Hsp70. Lamin B was detected for loading control.

**Figure 2:**
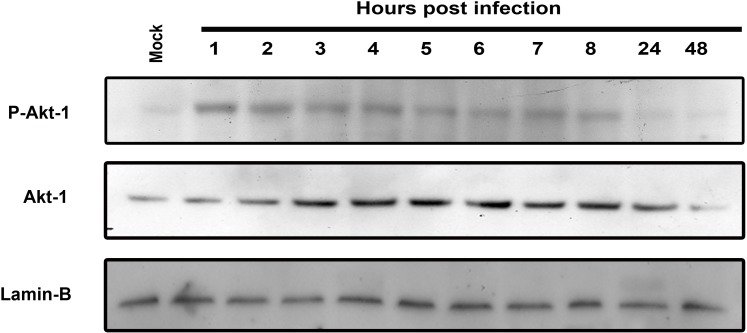
Activation of Akt-1 by phosphorylation in following VLP-treatment. CV-1 cells treated with 50 ng VLPs per 10^6^ cells were analyzed by Western blotting with anti P-Akt-1 and Akt-1 antibodies. Lamin B –loading control.

It was previously demonstrated that SV40 induces Hsc70 15–20 hours post infection, subsequent to genome entry into the nucleus [17], [18]. It was suggested that Hsc70 induction was a response to cell proliferation stimulus, presumably via the action of T-antigen. However, we found (**Figure 1A and B**) that Hsp/c70 upregulation does not require T-antigen, as it occurs in monkey kidney CV-1 cells in response to cellular uptake of VLPs alone, devoid of DNA and T-antigen. Confocal microscopy at 9 hours following the addition of 50 ng VLPs per 10^6^ cells (**Figure 1A**) showed increased level of Hsp/c70. Western analysis demonstrates that Hsp/c70 is upregulated already at 6 hours, with a peak at 12–24 hours (**Figure 1B**). Lamin B was used as loading control, since we found its level to be stable following SV40 or VLP-infection (unpublished). We further found that VLPs activated Akt-1 via phosphorylation within 1 hour (compare to mock-infected cells, **Figure 2**). Activation of Akt-1 continued for at least 8 hours after VLP addition. The level of Akt1 protein starts to increase at 3 hours post infection.

### Establishment of a pre-clinical model for AKI

**Figure 3:**
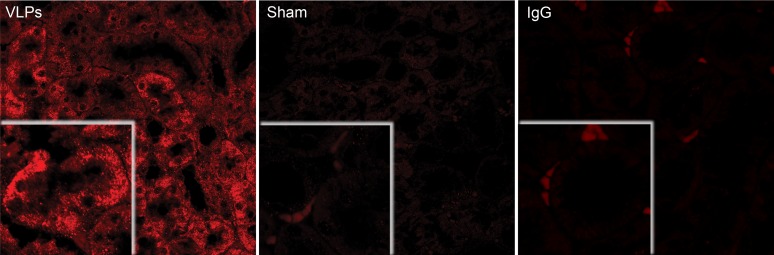
VLPs reach the kidneys. VP1 viewed by confocal microsocopy of kidney section harvested from a mouse treated with 0.1 mg/kg VLPs on 3 subsequent days, total 0.3 mg. VP1 was detected with polyclonal anti-VP1 antibody in renal tissue sections and viewed by confocal microscopy at magnification x60 and zoom x2 (shown in the inserts). *Left*–VLP-treated mouse. *Middle*–Sham mouse. *Right*-IgG control for non-specific staining by the secondary Cy5 conjugated antibody. The picture represents a kidney section of a VLP-treated mouse.

The findings described above suggested that if VLPs could target the kidneys *in vivo*, they might provide protection against AKI. We expected the VLPs to target the kidneys, as it is a natural environment of SV40 in primates and of the closely related BK virus in humans. Confocal microscopy demonstrated extensive and dense cytoplasmic staining for VLPs with anti-VP1 antibody in mice kidney sections, following tail-vein injections (**Figure 3**). We therefore set to establish an animal model for AKI.

Most animal models of renal failure are not highly reproducible and poorly represent clinical manifestations of this condition [19], [20]. Following preliminary experiments we chose to use the mercury intoxication model due to its robustness. Intraperitoneal injection of HgCl_2_ to mice led to elevated blood urea in a dose-dependent manner (**Figure S2**). For the studies shown below we selected a dose of 6.5 mg/kg. In our hands, mortality rate at this dose was ∼90% within 7 days.

### VLPs protect against mercury-induced cell death in a tissue-culture model

**Figure 4:**
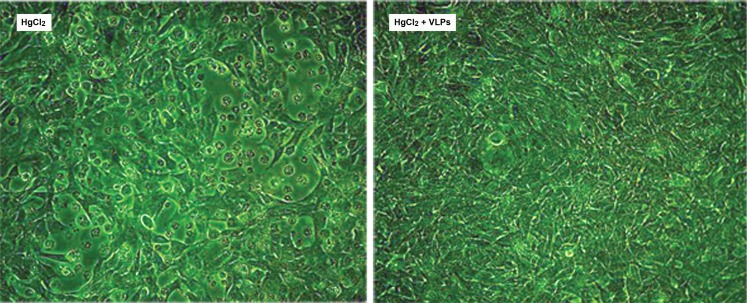
VLPs protect against HgCl_2_ induced death in MCT cells. *Left-*MCT cells treated by 15 µM HgCl_2_; *Right-*MCT cell pretreated with 50 ng/10^6^ cells for 4 hours before the addition of 15 µM HgCl_2_. Images were taken 3 days later. For clarification, both images were identically adjusted using Adobe Photoshop CS2, by ‘autolevel’ and by increasing brightness (+30).

Before beginning experiments in animals, the hypothesis that SV40 VLPs may protect against kidney damage was tested in cultured immortalized mouse tubular cells, MCT, [21], an *in vitro* system that best represents the target tissue. Exposure of the cells to 15 mM HgCl_2_ led to extensive cell killing, as seen by a large proportion of rounded, floating cells (**Figure 4** left). However, pretreatment with 50 ng VLPs/10^6^ cells, equivalent to an infection with SV40 at a multiplicity of 10 pfu (plaque forming units) per cell, provided effective protection (right). Supportive results were obtained for HEK293 and CV-1 cells (not shown). We also found that VLPs are capable of cell protection not only against HgCl_2_-induced damage, but also against other toxic agents, such as etoposide (see **Figure S3** for HEK293 cells).

**Figure 5:**
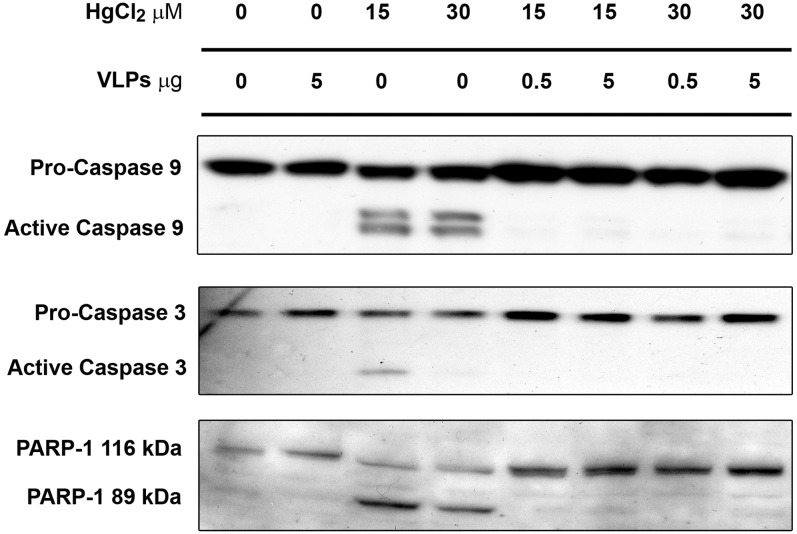
VLPs prevent activation of caspase 3 and 9 in HgCl_2_-treated MCT cells. Western blot analysis of extracts of MCT cell, treated as designated above. 50 µg of the total protein lysates were loaded on each lane. The antibodies used for detection are shown on the left.

In a similar experiment (**Figure 5** **)** MCT cells treated with HgCl_2_ with and without VLP-pretreatment, were analyzed for the activation of caspase 9 (as indicated by its autocleavage to 35 kDa) and activation of caspase 3 (auto-cleaved to 17 kDa fragment). The results presented in Figure 5 demonstrate that pretreatment with VLPs abolish the HgCl_2_-induced activation of both caspases. Similary, inhibition of caspase 3 activation was also indicated by elimination of the 89 kDa cleavage product of the apoptotic protein marker PARP-1.

### Pre-treatment with VLPs increases survival of AKI animals

**Figure 6:**
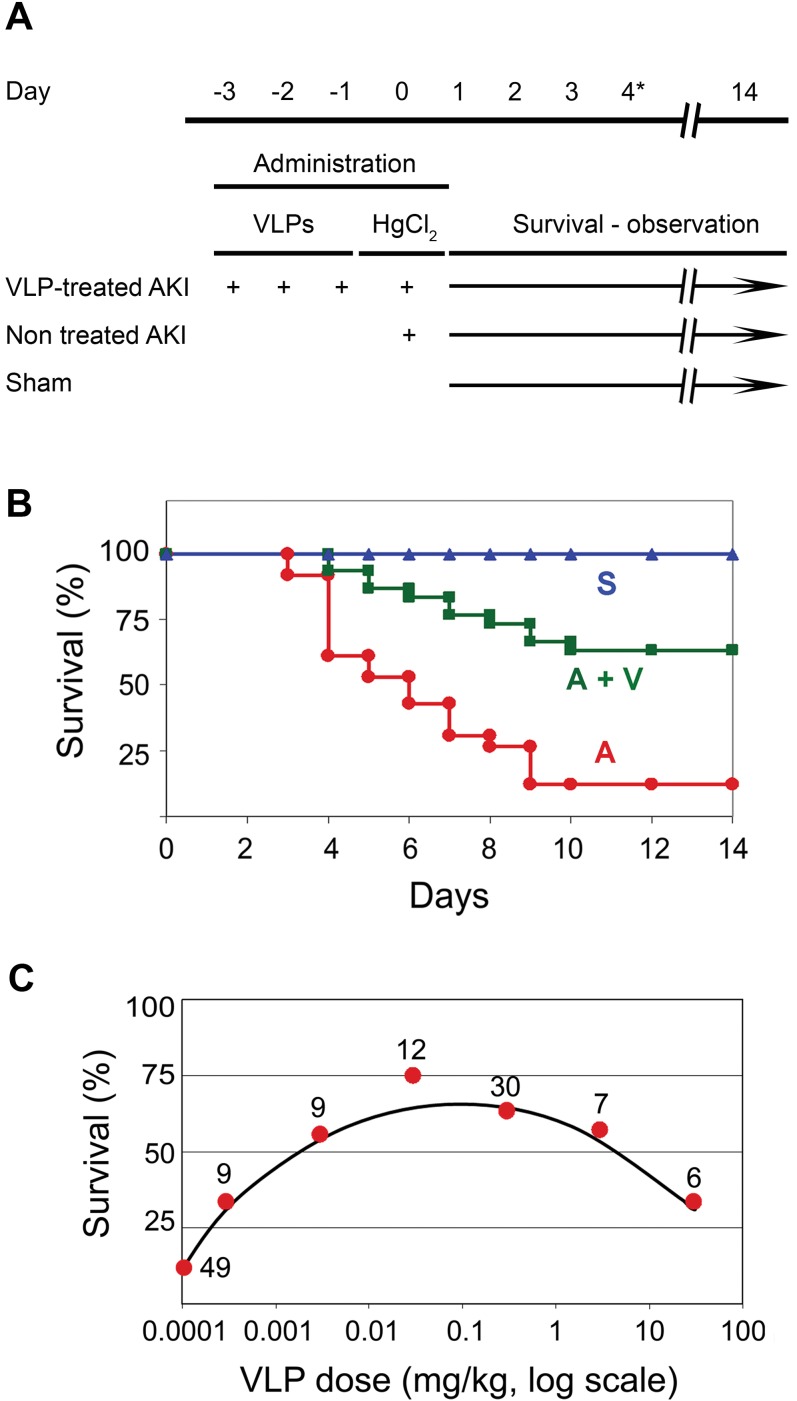
Effect of VLPs on survival rate of AKI mice. (A) Experimental design. (B) Survival of mice treated with 0.3 mg/kg VLPs and 6.5 mg/kg HgCl_2_. (c*)* Dose response. The number of mice treated at each VLP dose is shown above. The dose is presented on a logarithmic scale.

The critical question was whether VLPs are capable of protecting mice against HgCl_2_ insult. For the initial studies we used animal survival as an indication of protection. The design of the experiments is shown in **Figure 6A**. Animals were treated in 3 groups: Sham, non treated AKI mice and VLP-treated AKI animals. VLPs (0.3 mg/kg, in saline) were administered to mice via the tail vein in 3 consecutive daily injections (0.1 mg/kg each day, on days -3, -2, -1). Mice that did not receive VLPs were injected in parallel with the same volume of saline. HgCl_2_ in PBS was injected intraperitoneally on the fourth day, which was counted as day 0, to non-treated and to VLP-treated AKI animals. Sham animals received intraperitoneal injection of the same volume of PBS in parallel. Clinical parameters were evaluated on day 4 and survival was followed until day 14. Pre-treatment with VLPs resulted in an increase in survival rate from 12% (6/49) to 63% (19/30) (**Figure 6B**). Statistical analysis using Kaplan-Meier/ Mantel-Cox log-rank test indicated significance at P = 2×10^−6^. The hazard rate was 4.04 (95% CI: 2.07–7.91), meaning that death hazard for a mouse in the non-treated AKI group was 4.04 times greater than for a mouse in the VLP-treated group. Furthermore, survival correlated with a milder illness. Most of the animals in the AKI group were very sick, as seen by immobility, decreased appetite and alertness, in particular from day 3 onwards (compare **movie S2**–a typical AKI animal, to **movie S1**–sham animal). In contrast, many of the VLP-treated AKI animals exhibited only mild to moderate symptoms (**movie S3**) and started improving from day 3-4, reaching full recovery within a week. VLP-treated animals were thus protected from AKI-related morbidity and mortality. The attenuating effect was seen over a wide range of doses, from 3 µg/kg to 3 mg/kg (**Figure 6C**). Each dose was given in 3 aliquots, following the same experimental course as above (**Figure 6A**). The numbers above the points designate the number of mice tested at the particular dose. The lower survival rate at the highest VLP dose that was tested, 30 mg/kg, may be due to toxic effect of the protein.

### Pathological examinations and analyses of clinical parameters

**Figure 7:**
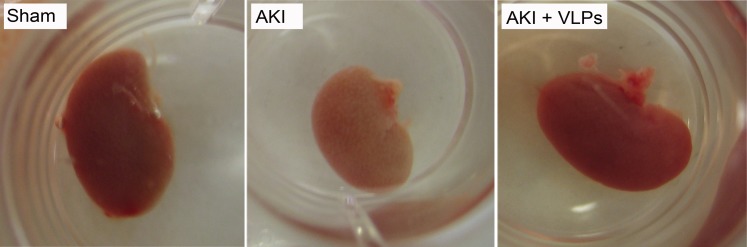
Gross appearance of kidneys. Mice were sacrificed 4 days post HgCl_2_ administration.

**Figure 8:**
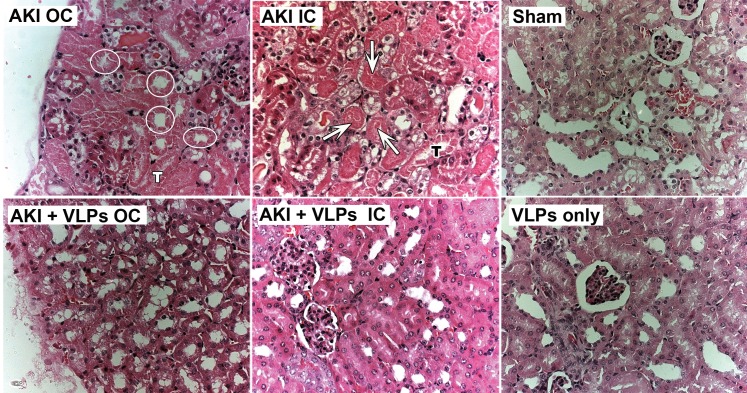
Kidney pathology at 4 days post HgCl_2_ administration. Sections of renal tissue stained with hematoxylin & eosin (magnificationX20). OC–outer cortex, IC–inner cortex. Note intratubular debris containing remnants of damaged tubular epithelial cells (arrows). Circles encompass tubuli lined by necrotic epithelial cells. Note lack of nuclei, granular eosinophilic cytoplasm and preserved cellular outline –‘tombstone‘ (T). As pictures were taken under variable lighting conditions, they were enhanced using ‘auto levels’ adjustment, Photoshop CS2 for Macintosh.

**Figure 9:**
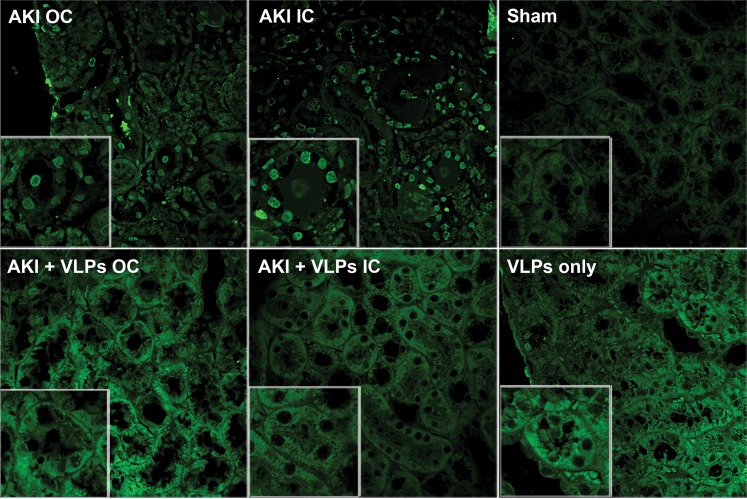
Detection of apoptosis in kidney sections. TUNEL assay was performed 4 days post HgCl_2_-treatment. Slides were photographed at x60 and Zoom 2 (see inserts). For positive and negative controls for the TUNEL assay see Figure S4.

Representative kidneys of the 3 groups of mice were harvested on day 4 following the insult (**Figure 7**). While the AKI kidneys were small and pale, kidneys from VLP-treated mice appeared macroscopically similar to sham kidneys. Histopathological examination of hematoxylin-eosin stained kidney sections (day 4, **Figure 8**) showed widespread necrosis of proximal tubular epithelial cells in the outer cortex (AKI OC). Note absence of nuclei in necrotic cells (circled tubuli), and shrinkage and detachment of necrotic epithelial cells from underlying tubular basement membrane, with ‘tombstone’ appearance (labeled ‘T’). The arrows point to massive debris in tubular lumen, composed of cellular fragments and proteinaceous material. In contrast, in VLP-treated mice apoptosis and necrosis were dramatically diminished, with only scant necrotic tubular cells, minimal tubular debris and maintenance of tubular morphology (**Figure 8**). The only abnormalities found were occasional tubular dilatation and irregular large cytoplasmic vacuoles, in particular in the outer cortex (**Figure 8** OC). Apoptosis was assayed by TUNEL staining, which detects intranuclear DNA damage (negative and positive controls for TUNEL staining are presented in **Figure S4**). Supporting the pathological picture, TUNEL detected extensive apoptosis of tubular cells of AKI animals (**Figure 9**, TUNEL positive nuclei), in particular in proximal convoluted tubuli, while very few TUNEL positive cells were seen in the VLP-treated animals (**Figure 9**, see magnified insert in OC). Notably, there was no indication that the VLPs induced inflammatory response during the first 4 days (see hematoxylin-eosin stained sections, **Figure 8**).

**Figure 10:**
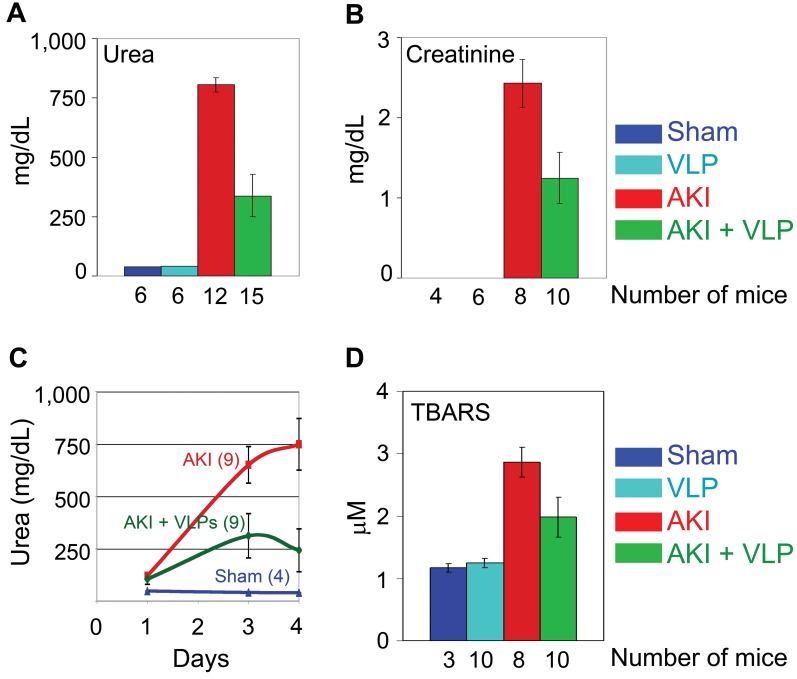
Serum indices: Serum urea level (A) and serum creatinine (B) 4 days following HgCl_2_ administration. S–sham mice; V–control mice treated with VLPs; A–AKI mice; A+V–AKI mice treated with VLPs. The number of mice in each column is depicted below. (C) Urea level measured at several time points after HgCl_2_ administration (at day 0). The number of mice in each group was: Sham–4 mice; AKI–9 mice; AKI+VLP–9 mice. (D) TBARS assays were performed 4 days post HgCl_2_ administration.

Serum urea and creatinine, the most commonly used clinical indices and prognostic indicators of renal function [22], [23] were also tested 4 days post insult. Both were markedly elevated in the non-treated AKI mice, signifying acute renal failure. This increase was significantly attenuated in the VLP-treated animals (**Figure 10A and B**), with P values 1×10^−4^ for urea and 0.016 for creatinine. Creatinine levels in sham animals and in mice treated with VLPs alone were below detection. While in the non-treated AKI animals serum urea increased continuously during the 4 days of the experiment, in the VLP-treated AKI mice serum urea reached a peak on day 3 and decreased thereafter (**Figure 10C**). This pattern is compatible with recovery of renal function and correlated with the higher survival rate of VLP-treated animals.

### Oxidative stress in VLP-treated and non-treated AKI mice

A hallmark of nephrotoxicity is oxidative and nitrosative stress as evident by overproduction of NO and reactive oxygen species [24], which most likely lead to tubular cell death [25]. Reactive hydroxy-free radicals *in vivo* may be evaluated via the level of lipid peroxidation, measured as thiobarbituric acid reactive substances (TBARS) [26]. As seen in **Figure 10D**, lipid peroxidation was significantly reduced in VLP-treated mice (P = 0.04) at day 4 post insult. We propose that attenuation of oxidative stress by the VLPs is part of the process of protection of renal function from mercury-induced nephrotoxicity.

### Activation of renal Akt-1 and upregulation of Hsp/c70 by VLPs *in vivo*

**Figure 11:**
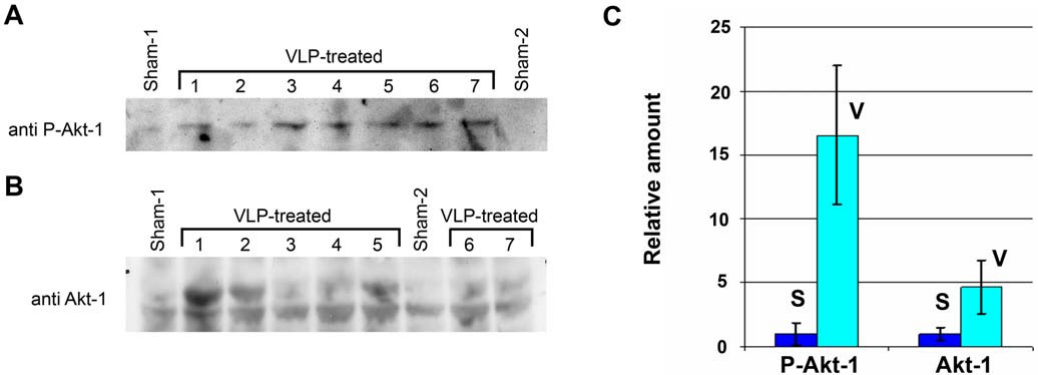
Activation of Akt-1 by VLPs in the kidney. (A) Ser 473 phosphorylated Akt-1 detected by anti-P-Akt antibody. (B) Detection of total Akt-1 by Akt-1 antibody. Total soluble proteins (10 µg for P-Akt-1 and 250 µg for total Akt-1) extracted from kidneys of 2 sham mice and 7 VLP-treated mice were loaded on each lane. The numbers above designate specific Sham and VLP-treated mice. (C) The levels of P-Akt-1 and Akt-1 in VLP-treated (V-7 mice)) and sham (S-2 mice). Evaluation was performed by TotalLab software.

The results led us to hypothesize that renal protection is mediated via P-Akt-1 and Hsp/c70. We therefore tested whether VLPs activated both pathways *in vivo*. For effective protection, both activities are probably required at the onset of the mercury insult, day 0 in our experimental scheme. Mice were injected with 0.3 mg/kg VLPs for 3 consecutive days (as in **Figure 6A**) and their kidneys were harvested on the 4th day (day 0). Total kidney proteins were extracted and analyzed by Western blotting. Figure 11A demonstrates activation of Akt-1 by serine 473 phosphorylation for all 7 mice tested, while in sham mice phospho-Akt-1 was hardly detectable. Similar analysis on the same 9 mice (**Figure 11B**, 7 experimental and 2 controls, loaded in a different order) using anti-Akt-1 antibodies, shows both the phosphorylated and the non phosphorylated Akt-1 proteins. The level of non-phosphorylated Akt-1 was almost unchanged. Thus total Akt-1 protein in the VLP-treated animals was increased, indicating upregulataion.

Evaluation of the bands (**Figure 11C**) showed that the level of phospho-Akt-1 was elevated ∼15 fold, while total Akt-1 increased by ∼5 fold.

**Figure 12:**
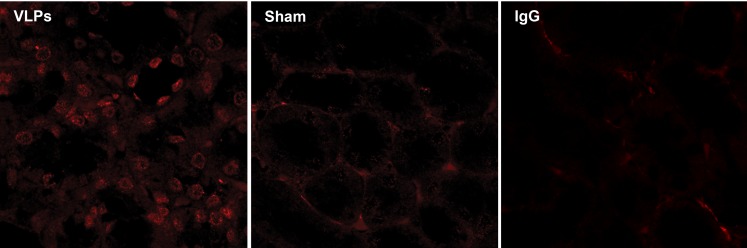
Upregulation of Hspc/70. Hsp/c70 viewed by confocal microscopy at magnification x60 and zoom x2. *Left*–VLP-treated mice. *Right*–Sham mice. IgG-negative control. For clarification, both images were identically adjusted using Adobe Photoshop CS2, by ‘autocolor’ adjustment.

Upregulation of Hsp/c70 was detected by confocal microscopy of kidney sections. **Figure 12** shows a typical staining pattern. While tubular cells of sham animals showed faint positive immunostaining for Hsp/c70, the VLP-treated animals had a markedly higher signal, appearing as a speckled ring around the nucleus, demonstrating significant upregulation. Non specific staining by the Cy5 conjugated anti mouse antibodies is shown in the IgG control.

## Discussion

The present study shows that SV40 VLPs, devoid of DNA, protect animals from mercury-induced AKI as demonstrated by attenuation of renal failure, seen by lower serum creatinine and urea, and by dramatic increase in survival. Tubular cell injury and apoptosis were almost eliminated. Our results suggest that renal protection is at least partly achieved by decrease in oxidative stress. The wide dose range suggests that VLPs are not toxic at the levels required for kidney protection. Furthermore, there was no indication for inflammatory response following VLP administration.

Our study demonstrates dramatic activation of Akt-1 by VLPs, with moderate upregulation of the protein. Furthermore, the level of Hsp/c70 is greatly increased. Notably, the timing of elevation of Ser 473 Phospho-Akt-1 (the activated species) and Hsp/c70 coincided with the time when animals in the survival study were given the mercury insult. Furthermore, the immunoblot analysis of Akt-1 and confocal microscopy of Hsp/c70 of additional animals suggest that variation in the degree of activation between the different animals was low. Thus the mechanism of protection by VLPs against AKI is based on activation of both pathways leading to survival. Chaperons were recently implicated in renal protection against AKI [7], [8], [9], [10]. Akt-1 is a key player in activation of survival pathway, leading to apoptosis arrest [27], [28]. Akt-1 was also shown to diminish oxidative stress [29]. Our experiments in cultured mouse tubular cells indicate that part of the apoptotic arrest is mediated via the inhibition of capspase 9 and caspase 3.

Our ongoing studies in cultured monkey kidney cells (Butin-Israeli and Oppenheim, unpublished), using proteomics arrays, have indicated that VLPs activate PLCγ. Using specific inhibitors we found that Akt-1 activation fully depends on PLC-signaling, and only partly on PI3K. Activation of Akt-1 by phosphrylation at serine 473 was shown to lead to survival pathway [30] via regulation of proapoptotic proteins such as Bad, caspase 9 and p53 [28]. Furthermore, a number of publications showed that activation of PLC-PI3K-AKT signaling led to apoptosis arrest in neurodegeneration [31] and atherosclerosis [32]. We propose that a similar pathway is induced by the SV40 VLPs in the kidney. Our unpublished studies further indicate that PLCγ signaling leads to upregulation of Hsp/c70. Induction of Hsp/c70 might proceed via PLC dependent PKC activation as previously described for a heat stress model [33].

Chaperones play a role in SV40 disassembly [34]. Here we show that the infecting virus induces chaperone upregulation via signals triggered by its coat proteins, very early post infection. Taken together, these findings suggest that upregulation of cellular chaperone may be part of the signaling program that functions in viral entry and disassembly.

We have asked whether VLPs may also have a therapeutic effect for animals with AKI. The experiments indicated that administration of VLPs following the HgCl_2_ insult did not lead to increased survival. This is most likely because of the time required for the induction of the Akt survival pathway and chaperone upregulation. Thus, the VLPs do not appear to lead to kidney regeneration, but rather to prevention of injury.

A critical limitation in treating human patients with a foreign protein such as SV40 VLPs is the risk of developing immune response, which would preclude repeated administrations. We and others found that SV40 vectors used for gene delivery in mice did not elicit neither cellular immune response [35] nor humoral immune response [35], [36]. This may be partly due to the unique mode of entry of SV40 via caveolae and the ER, bypassing the endosomal pathway [3]. In addition, we found that SV40 activates FLIP, via phosphorylation, following infection of tissue-cultured cells. FLIP was demonstrated to suppresses downstream signals from T killer cells [37] and could therefore also partly account for non-immunogenicity of SV40 vectors. The early activation of FLIP (within one hour of SV40 infection, long before the viral genome enters the nucleus and starts transcribing) suggests that it would also be induced by VLPs. We therefore anticipate that like SV40 vectors, VLPs might also be non-immunogenic, allowing repeated treatments. Of note, FLIP activation by SV40 may be another anti-apoptotic renal protective mechanism, in addition to AKT activation and Hsp70 upregulation.

In this proof-of-principle therapeutic study we showed that VLP administration conferred protection against HgCl_2_-induced AKI, presumably via activation of the Akt-1 survival pathway and chaperones. Production of VLPs is a simple, low cost procedure, and the absence of genetic material circumvents inherent risks of gene therapy. Thus this strategy may potentially be developed as a preventative medical measure against AKI.

VLPs protected human HEK293 and monkey CV-1 cells also against etoposide-induced apoptosis, suggesting wide range applicability of VLPs for cell and tissue survival. SV40-based vectors were shown to reach many targets in vivo [38], [39], [40]. Numerous reports suggest that Akt survival pathway and chaperones are involved in many types of organ failure, not just the kidney. Thus the findings described here may be extended to additional clinical conditions. Furthermore, viruses have evolved to enter cells by a variety of mechanisms, inducing a wide array of cellular responses. One may predict that virus-induced host signaling would by applicable as novel medical modalities to a large spectrum of diseases.

## Materials and Methods

### Cell cultures and media

*Spodoptera frugiperda* (Sf9) cells were grown at 27°C in serum-free Bio-insect medium containing glutamine, penicillin, streptomycin and amphotericin (Biological Industries, Israel). African Green monkey CV-1 cells (ATCC # CCL-70), Human Embryonic Kidney 293 cells (ATCC #CRL-1573) and Mouse Tubular cells [21] were cultured in high glucose Dulbecco's modified Eagle's medium containing glutamine, penicillin, streptomycin, and 10% FBS.

### Production and purification of VLPs

Recombinant baculovirus expressing VP1 (Swiss-Prot P03087, PDB 1SVA) from the polyhedrin promoter were used for production of VLPs as previously described [15]. VLPs were purified by either of two methods, as described below. Both methods gave essentially the same results.

In the first set of experiments we used a method developed for assembly of VP1 around DNA, except that DNA was omitted from the assembly reaction [41]. VP1 was isolated from nuclear extracts of Sf9 cells, containing 1–3 mg VP1/ml nuclear extract. The VLPs were purified and concentrated by stirred-ultrafiltration under Argon using XM300 membrane (Millipore). They were stored in 0.5 N NaCl and diluted to isotonic ionic strength immediately before use.

In later experiments the VLPs were harvested from the medium of baculovirus-infected Sf9 following cell lysis, 5 days post infection, as follows: intact cells and cell debris were removed by centrifugations at 6,000xg for 10 min. The supernatant was further clarified at 17,000xg for 20 min. VLPs were precipitated at 80,000xg, 3 hrs. The VLP pellet was suspended in 0.5 M NaCl, purified by ultrafiltration and stored as above. As stated above both methods of preparation gave essentially the same results.

Total protein was determined by the Bradford assay. The amount and purity of VP1 in each VLP preparation was estimated by SDS-PAGE and staining with Coomassie or Silver Stain. Both staining methods gave similar results.

### Animal studies

Balb/C female mice, 8–10 weeks, 19–21 gm, were kept in a SPF unit in the animal facility at the Hebrew University Medical School. VLPs were injected via the tail vein in a total volume of 0.1 ml. HgCl_2_ was injected intraperitoneally in a total volume of 0.4 ml. Mice were sacrificed at the indicated times humanely via cervical dislocation under deep anesthesia. Animal care and experiments were conducted in accordance with the Hebrew University-Hadassah Animal Authority guidelines.

### Serum tests

Serum urea and creatinine were measured using Reflotron sticks (Roche). TBARS assay was performed according to the protocol of Animal Models of Diabetic Complications Consortium (AMDCC; www.amdcc.org/shared/Protocols.aspx).

### Protein expression studies

Cultured cells: VLPs were added to subconfluent cells, in a small volume of serum-free medium, grown in 25 cm^2^ tissue-culture bottles (for total protein extraction and Western analyses) or on microscopic cover slips (for phase and confocal microscopy). 50 ng VLPs were added per 10^6^ cells, approximately equivalent to multiplicity of infection (moi) of 10 plaque forming units (pfu) of SV40 per cell. (Molecular weight of a VP1 capsid is ∼15 MDa, thus 50 ng represent 2×10^9^ capsids. Not more than 1 of 200 particles in SV40 stocks are infectious [42] (and our unpublished data), 2×10^9^ SV40 virions contain 1×10^7^ pfu, or moi 10 per when applied to 10^6^ cells.

The VLPs were allowed to adsorb to the cells in the cold on a gyratory shaker, at 20 rpm. Medium was added and the cells were transferred for incubation at 37°C. Total protein extracts were prepared by lysing the cells in 0.6% SDS in 10 mM Tris pH-7.4 buffer. Samples containing 50 µg protein were loaded on each lane. Hsp/c70 was detected by confocal microscopy in cells fixed in 4% formaldehyde.

Mice kidneys: Proteins were extracted from frozen mouse kidneys using RIPA [43] For microscopy, kidneys were fixed in 4% formaldehyde. Paraffin-embedded kidney sections were dewaxed, rehydrated and stained.

### Antibodies

The following antibodies were used: Akt-1, polyclonal; P-Akt-1, polyclonal against phosphorylated S473; Caspase 9, monoclonal; Caspase 3 polyclonal (Cell Signaling). Hsp/c70, monoclonal (clone BB70 Stressgen); PARP-1, polyclonal antibody (Alexis). Lamin B, polyclonal antibody (Santa Cruz). VP1, polyclonal antibody prepared in our laboratory [15]. For confocal microscopy we used Cy5 as secondary antibody. Controls for confocal microscopy included sections without any staining, sections stained with secondary antibody only, and sections obtained from sham animals.

### TUNEL detection

DNA strands breaks detected by TUNEL, using In Situ Cell Death Detection kit, and fluorescien (Roche), following manufacturer protocol. Briefly, paraffin-embedded kidney sections harvested on day 4 were dewaxed, rehydrated and protease-treated (10 mg/ml proteinase K, Roche), followed by permeabilization by Tritone X-100, labeling with terminal transferase and detection by confocal microscopy. Negative control slides were incubated with the same labeling solution without terminal transferase. Positive control slides were prepared by adding DNAse I (5 u/ml) for 1 hr at room temperature after permeabilisation.

### Statistical analysis

Survival data were analyzed statistically using the Kaplan-Meier procedure, and the Mantel-Cox log-rank test was applied for comparing survival curves between study groups. Quantitative clinical indices were analyzed by comparing the VLP-treated and untreated AKI groups using Student's independent t-test (two-tailed). A P-value of 5% or less was considered statistically significant.

## Supporting Information

Figure S1 Structure of the VLPs. Transmission electron microscopy pictures of VLPs and wild type SV40. Samples, adsorbed onto UV-irradiated formovar-carbon-coated copper grids and stained with 1% uranyl acetate. The samples were viewed in a Philips CM-12 electron microscope, using a voltage of 100 kV, and photographed at x53,000. The bars represent 50 nm.
(1.96 MB TIF)

Figure S2 Nephrotoxic mouse model for AKI. HgCl2 was injected intraperitoneally (on day 0) in a total volume of ∼0.4 ml to 9–10 weeks old female Balb/c mice. Average weight ∼20 gr. Blood was withdrawn from the tail vein and urea level was measure in the serum.
(2.35 MB TIF)

Figure S3 Protection of HEK293 from etoposide-induced apoptosis by VLPs. 50 µM etoposide was added to HEK293following pretreatment with 50 ng/106 cells VLPs for 4 hours. Images were taken 3 days later.
(4.24 MB TIF)

Figure S4 Controls for TUNEL staining. (A). Negative controls: Mouse tissue was treated as for TUNEL staining but without terminal transferase. (B). Positive controls: Following permeabilization with Tritone X-100 the slides were treated with DNase I, then incubated with the complete labeling solution.
(9.93 MB TIF)

Movie S1 Sham mouse
(6.83 MB MOV)

Movie S2 AKI mouse
(8.84 MB MOV)

Movie S3 VLP-treated AKI mouse
(5.34 MB MOV)
