# Supplementary figures and images for: DNA-Free Recombinant SV40 Capsids Protect Mice from Acute Renal Failure by Inducing Stress Response, Survival Pathway and Apoptotic Arrest
Source: PLoS One. 2008 Aug 20;3(8):e2998. doi: 10.1371/journal.pone.0002998 (PMC2515219; doi:10.1371/journal.pone.0002998)

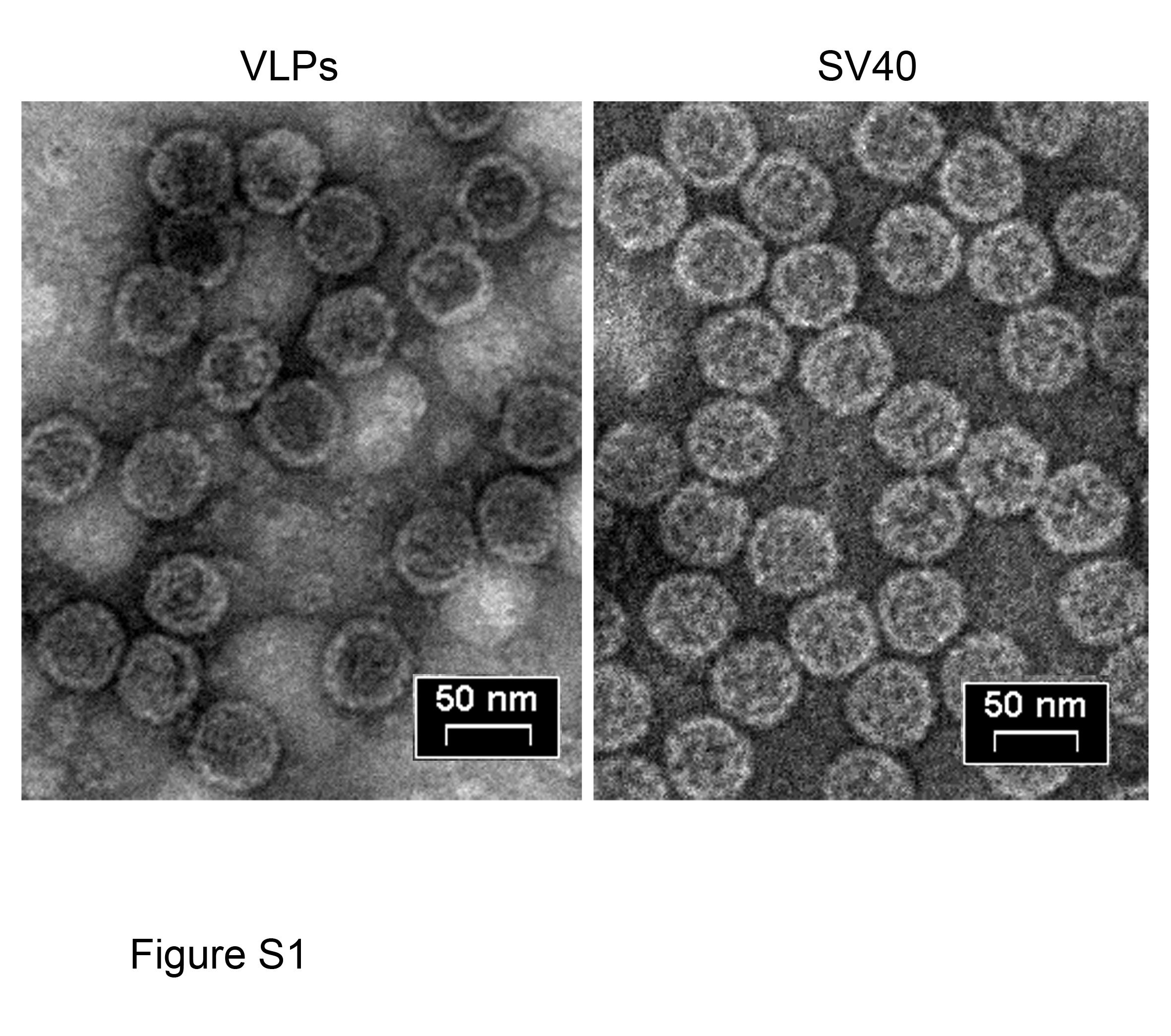

Supplement: Figure S1 — Structure of the VLPs. Transmission electron microscopy pictures of VLPs and wild type SV40. Samples, adsorbed onto UV-irradiated formovar-carbon-coated copper grids and stained with 1% uranyl acetate. The samples were viewed in a Philips CM-12 electron microscope, using a voltage of 100 kV, and photographed at x53,000. The bars represent 50 nm. (1.96 MB TIF) [file pone.0002998.s001.tif]

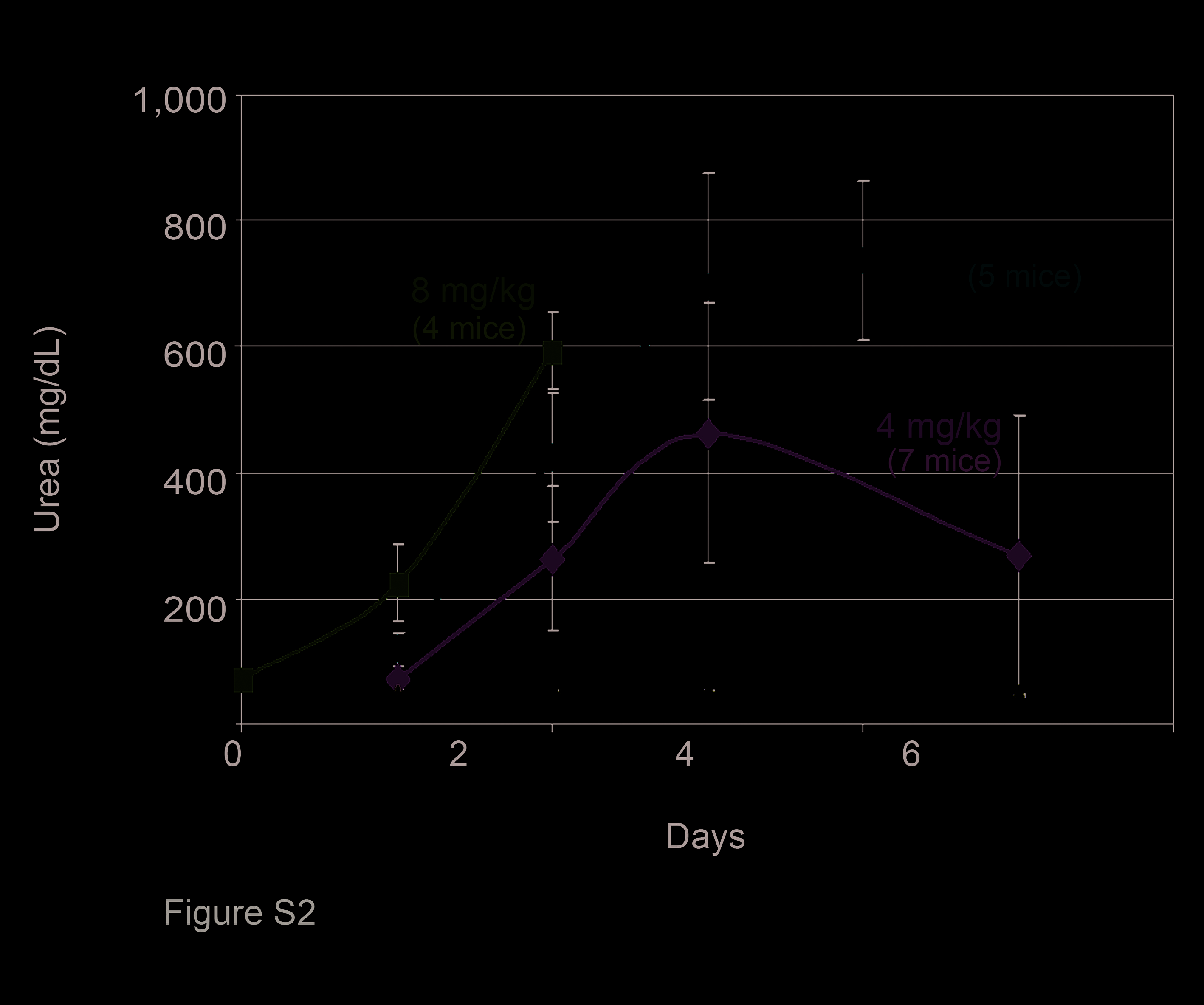

Supplement: Figure S2 — Nephrotoxic mouse model for AKI. HgCl2 was injected intraperitoneally (on day 0) in a total volume of ∼0.4 ml to 9–10 weeks old female Balb/c mice. Average weight ∼20 gr. Blood was withdrawn from the tail vein and urea level was measure in the serum. (2.35 MB TIF) [file pone.0002998.s002.tif]

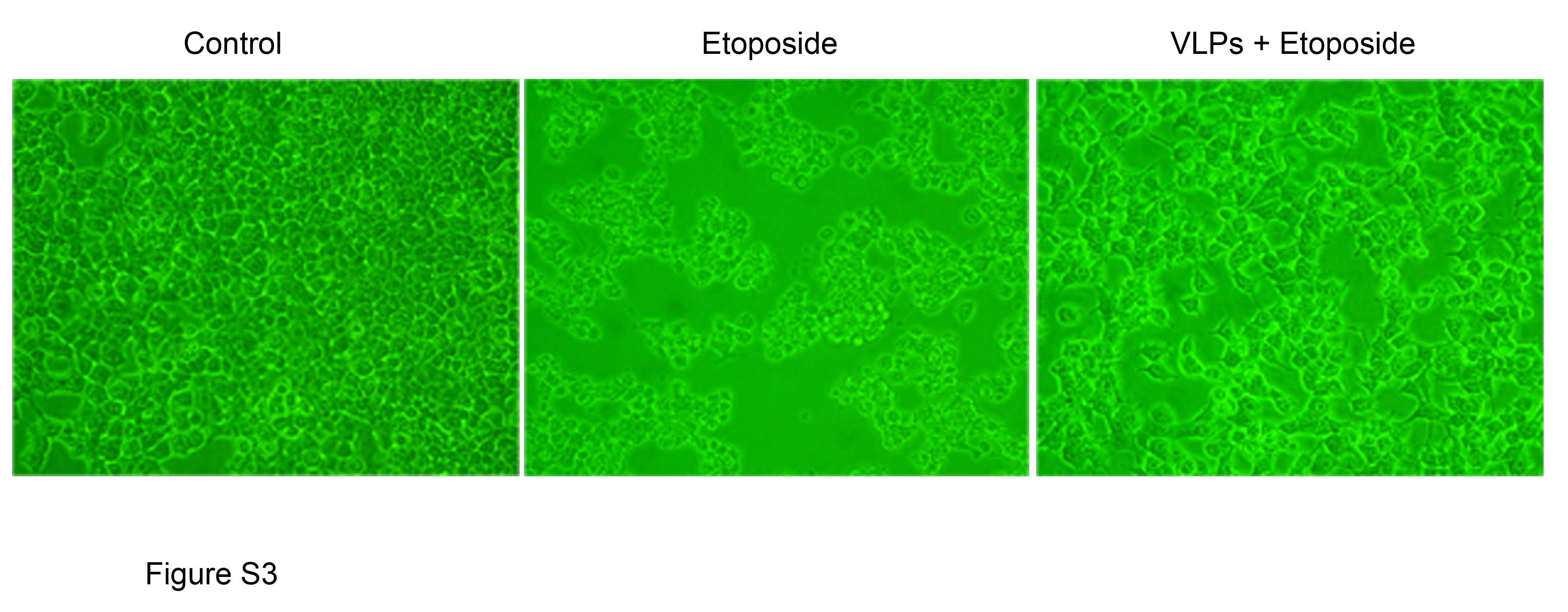

Supplement: Figure S3 — Protection of HEK293 from etoposide-induced apoptosis by VLPs. 50 µM etoposide was added to HEK293following pretreatment with 50 ng/106 cells VLPs for 4 hours. Images were taken 3 days later. (4.24 MB TIF) [file pone.0002998.s003.tif]

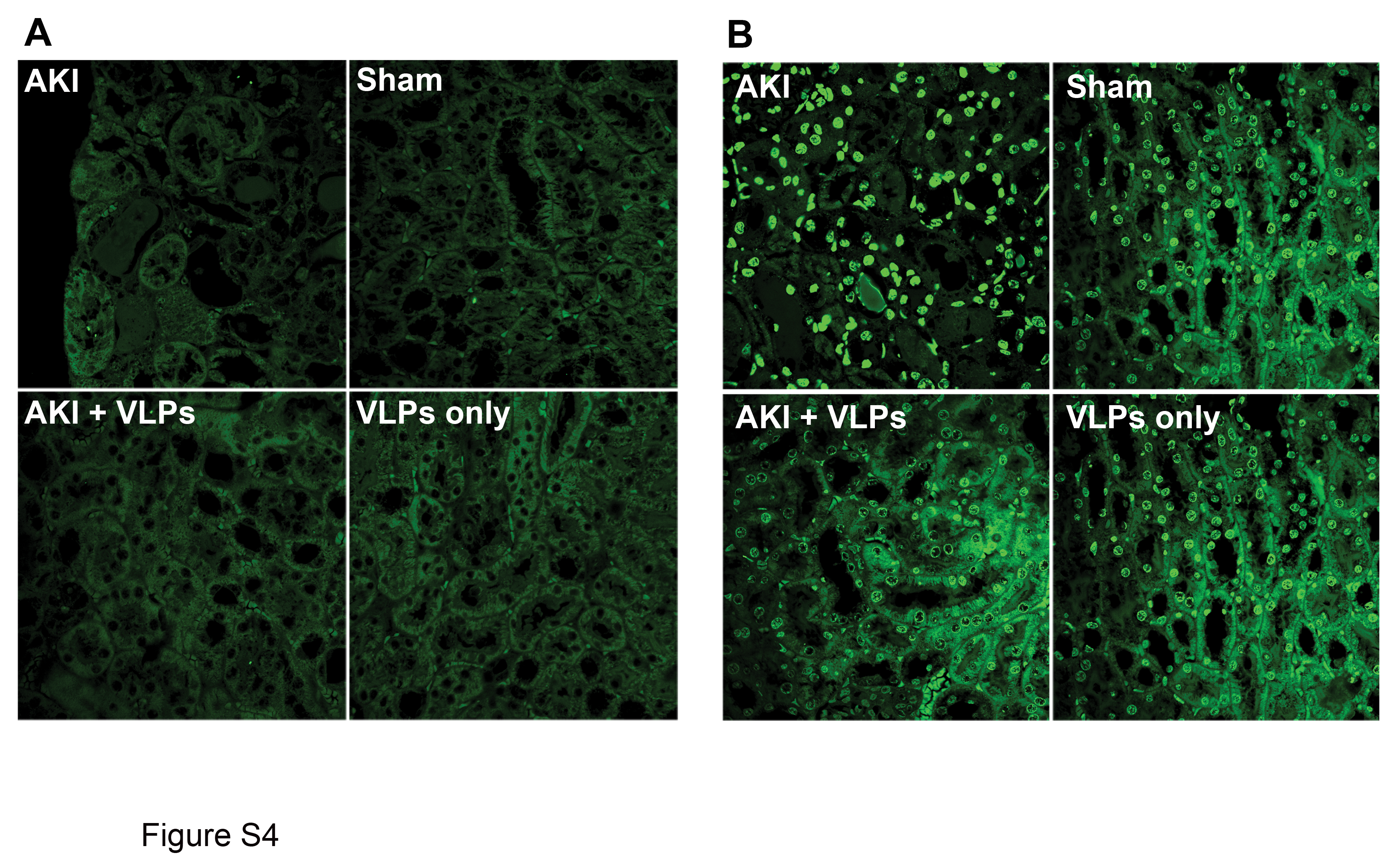

Supplement: Figure S4 — Controls for TUNEL staining. (A). Negative controls: Mouse tissue was treated as for TUNEL staining but without terminal transferase. (B). Positive controls: Following permeabilization with Tritone X-100 the slides were treated with DNase I, then incubated with the complete labeling solution. (9.93 MB TIF) [file pone.0002998.s004.tif]
